# A causal loop diagram of older persons’ emergency department visits and interactions of its contributing factors: a group model building approach

**DOI:** 10.1007/s41999-023-00816-8

**Published:** 2023-06-30

**Authors:** Oscar S. Smeekes, Hanna C. Willems, Ilse Blomberg, Bianca M. Buurman

**Affiliations:** 1 Internal Medicine, Section of Geriatric Medicine, Amsterdam UMC Location University of Amsterdam, Meibergdreef 9, 1105 AZ Amsterdam, The Netherlands; 2 Medicine for Older People, Amsterdam UMC Location Vrije Universiteit Amsterdam, Amsterdam Public Health Research Institute, De Boelelaan 117, Amsterdam, The Netherlands

**Keywords:** Causal loop diagram, Emergency department visits, Older persons, Acute care demand, Contributing factors, System dynamics

## Abstract

**Purpose:**

Understanding the etiology of older persons’ emergency department (ED) visits is highly needed. Many contributing factors have been identified, however, the role their interactions play remains unclear. Causal loop diagrams (CLDs), as conceptual models, can visualize these interactions and therefore may elucidate their role. This study aimed to better understand why people older than 65 years of age visit the ED in Amsterdam by capturing the interactions of contributing factors as perceived by an expert group in a CLD through group model building (GMB).

**Methods:**

Six qualitative online focus group like sessions, known as GMB, were conducted with a purposefully recruited interdisciplinary expert group of nine that resulted in a CLD that depicted their shared view.

**Results:**

The CLD included four direct contributing factors, 29 underlying factors, 66 relations between factors and 18 feedback loops. The direct factors included, ‘acute event’, ‘frailty’, ‘functioning of the healthcare professional’ and ‘availability of alternatives for the ED’. All direct factors showed direct as well as indirect contribution to older persons’ ED visits in the CLD through interaction.

**Conclusion:**

Functioning of the healthcare professional and availability of alternatives for the ED were considered pivotal factors, together with frailty and acute event. These factors, as well as many underlying factors, showed extensive interaction in the CLD, thereby contributing directly and indirectly to older persons’ ED visits. This study helps to better understand the etiology of older persons’ ED visits and in specific the way contributing factors interact. Furthermore, its CLD can help to find solutions for the increasing numbers of older adults in the ED.

**Supplementary Information:**

The online version contains supplementary material available at 10.1007/s41999-023-00816-8.

## Key summary points

**Aim:**

This study aimed to better understand why people older than 65 years of age visit the ED in Amsterdam and capture the interactions of contributing factors in a causal loop diagram (CLD) through group model building (GMB).

**Findings:**

Central contributing factors included, ‘acute event’, ‘frailty’, ‘functioning of the healthcare professional’ and ‘availability of alternatives for the ED’. These factors, as well as many underlying factors, showed extensive interaction, thereby contributing directly and via each other to older persons’ ED visits.

**Message:**

This study helps to better understand the etiology of older persons’ ED visits and elucidates the role interactions of contributing factors can play.

**Supplementary Information:**

The online version contains supplementary material available at 10.1007/s41999-023-00816-8.

## Introduction

An aging population accompanied by increased multimorbidity attributes to increasing emergency department (ED) visits of older persons. This is an urgent international concern causing burden at patient, caregiver, healthcare system and societal level [1, 2]. In the Netherlands, annually approximately 800,000 people aged over 65 visit Dutch ED’s [3]. Prognoses state that by 2030 ED visits will be up by 40%, while dealing with a decreasing ratio of healthcare workers in society [3].Therefore, it is paramount to develop a clear understanding of the key mechanisms that explain why older persons visit the ED and to explore effective interventions in the near future.

Multiple factors have been associated with older persons’ ED visits. A British nation-wide mixed methods study showed that socio-economic deprivation, poor access to primary care and availability of alternative care options out of hours, besides previous ED visits, geriatric conditions and multimorbidity result in higher risk for visiting the ED [4]. Furthermore, they reported that the complexity of ED visits was a result of personal, service and system interplay especially in the socio-economic deprived. Concerning ED return visits, a recent systematic review identified these same factors as important contributing factors [5]. Interestingly, in the Netherlands two root cause analyses of ED return visits concluded that disease-related factors account for about 50% of presentations [6, 7]. Human factors (19%), such as misdiagnosis by the consulted physician, and organizational factors (15%), such as unavailability of timely home care, contributed significantly [6]. An in-depth interview study on older persons’ perspectives identified the feeling of a crisis, the feeling that their general practitioner could not answer their care demand, incomplete discharge information at the ED, inadequate follow-up and lack of recovery after an ED visit, as contributing factors [8]. In addition, frail older persons identified the complexity of healthcare structure as contributing to an ED visit [9]. These studies show that older persons visit the ED not only because of acute disease, but also because of human factors, organizational factors, subacute disease as well as specific geriatric conditions which are often better managed elsewhere and could potentially be prevented. Furthermore, despite their valuable results, these studies have not elucidated how these contributing factors to older persons’ ED visits interact.

Both clinical practice as research have stated that understanding the interaction of contributing factors in the development of disease and illness behavior in older persons is essential for understanding their etiology, because they can often not be explained by a single factor or a few interactions [10–12]. This could explain why previous interventions for preventing older persons’ ED visits, targeting only single factors or some interactions, have not yet been effective [13–15]. To determine which prevention strategies have the potential to be effective, overview of key contributing interactions is needed [16].

Methods, such as group model building (GMB) and causal loop diagrams (CLD’s), from the field of system dynamics, can provide overview of a problems’ key contributing interactions [17]. In contrast to traditional research methods, they achieve such overview by approaching problems in a nonlinear way and by capturing feedback mechanisms as well as the total coherence of contributing factors in a conceptual model [17]. The model can help to develop effective interventions. A CLD is a model type that provides overview and is often the first step in system dynamics modeling of a problem [17, 18]. GMB is a participatory study design that lets participants combine their knowledge to build a model to represent their shared view on a problems’ etiology [17, 19]. Despite their potential, these methods have not been used to elucidate the key mechanisms that can explain why older persons visit the ED. To address these gaps, this study aimed to better understand why people older than 65 years of age visit the ED in Amsterdam by capturing the interactions of contributing factors as perceived by an expert group in a CLD through group model building (GMB).

## Methods

### Design

We used an online focus group like study design, known as group model building, to capture the views of a local interdisciplinary expert group on why people older than 65 years of age visit the ED in Amsterdam (see Appendix 1). In group model building, researchers facilitate specifically guided group discussion through use of scripts [20, 21]. These scripts provide specific activities and questions for the participant group to optimally discuss and capture their shared view on a problems’ etiology [20, 21]. In this study their views were described in a conceptual model, known as a causal loop diagram. The causal loop diagram depicted the year 2019 to exclude the effects of the COVID-19 pandemic. We conducted six GMB sessions between February and May 2021. These sessions were done online because of COVID-19 restrictions. OS and IB both designed as well as facilitated the GMB sessions, HW facilitated the GMB sessions and clarified output, and ER gave expert advice on design. Further information on the researchers’ backgrounds can be found in the background information on researchers section at the end of this paper. Sessions one to four formed the core of the GMB process and focused on clear description of most important contributing factors and their interactions. These sessions lasted 1.5 h, consisted primarily of validated GMB scripts from Scriptapedia (an open access online book containing guidelines and scripts for evidence-based GMB [22]) and were adapted to the online format. A detailed description of the GMB method used for this study as well as specific implementational challenges for this geriatric case were discussed in a separate article [23]. Sessions five and six were short non scripted sessions, used to clarify and validate the CLD with the interdisciplinary expert group.

### Causal loop diagram background

**Fig. 1:**
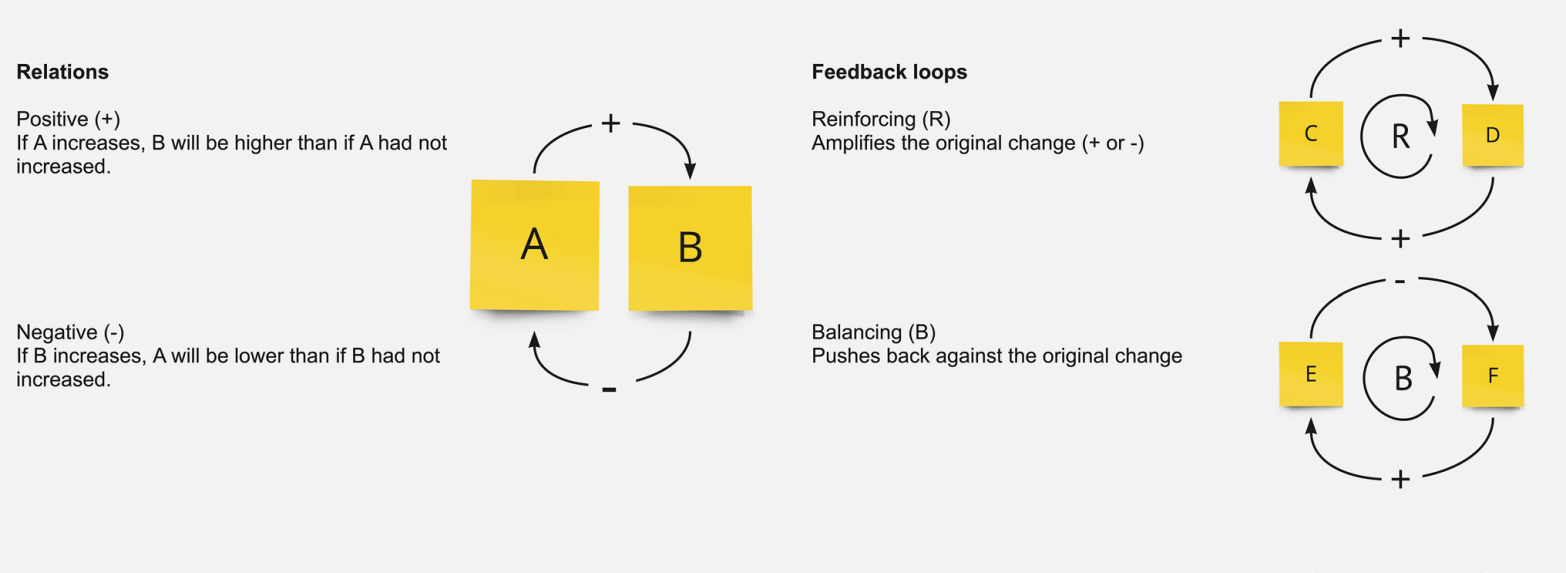
Relations and feedback loops in a causal loop diagram

A CLD is a visual representation of a problem’s assumed key causal factors and their key causal relations in its simplest form [17, 24, 25]. The simplest form meaning, that the CLD aims to depict the etiology of a problem with as little factors and relations as possible, as clearly as possible. As a result of GMB, it depicts a participant group’s shared view. A CLD consists of factors and arrows drawn between them. The arrows represent a positive (+) or negative (−) perceived causal relation by participants. A positive causal relationship between factor A and B for example, means that if A increases, B will also increase [17]. This is illustrated in Fig. 1. When a factor influences another factor or outcome, this can be done directly or indirectly (through another factor). Factors that have a “direct” effect on the outcome (older persons’ ED visits) are described as direct factors in the results of this study. Underlying factors that have an “indirect” effect on the outcome, are described as secondary or tertiary factors. Therefore, in CLD’s, causal relations can both be a result of interaction between factors as well as effect modification of one factor to another. For the legibility of our paper, we have chosen to use the term interaction to describe both effects. When multiple factors form a closed loop via their causal relations, a feedback loop is present. A reinforcing loop has a spiraling effect on the original change, and can push a system out of balance, where a balancing loop antagonizes the original effect and stabilizes the system. A reinforcing loop including factor C and D, for example, means that if C increases, C will increase further via D. This is illustrated in Fig. 1. Feedback loops can be important for understanding the amplification of inter-individual differences in risk profiles as well as identifying potentially effective intervening points [10, 17] and thus in development of older persons’ ED visits. After the construction of a CLD model, it is quantified and validated on both literature as well as historical data, to a more computerized model that represents reality in more detail and provides the possibility of simulation [26].

### Expert selection and recruitment

**Fig. 2:**
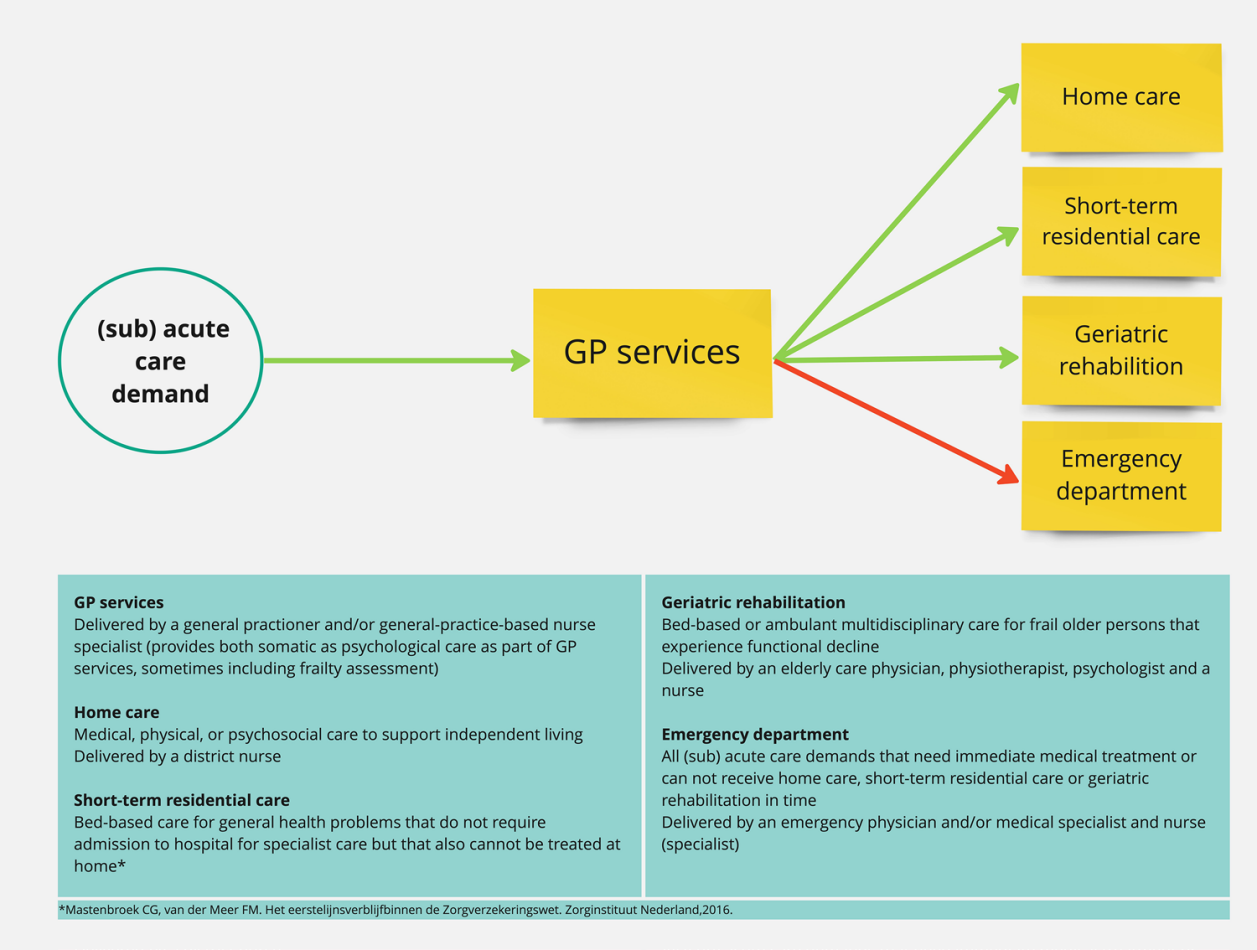
Overview of services in primary care, that are in operation in the Netherlands to prevent ED visits

Potential experts included local clinical and research experts on the older persons’ patient journey leading to ED visits in Amsterdam. An expert was defined as a key stakeholder in this journey, who was seen as an expert by colleagues and had at least 5 years of job experience. To gain the needed variety of viewpoints, while accounting for optimal discussion group size, to achieve the research aim, we purposefully sampled experts based on a predetermined essential profiles list. This list was based on our clinical and research experience. We identified nine essential profiles including a district nurse, ED physician, general practitioner (trained in elderly care), geriatrician, elderly care physician, nurse specialist geriatrics, nurse transfer coordinator, data analyst healthcare insurer, and a patient representative. A maximum of one expert per matching profile was included to secure optimal discussion group size as recommended by ER, and Wilkerson et al. [27]. Inclusion criteria were: meeting expert criteria in one or more of nine profiles as described in Table 1, motivation to participate in the full process, and availability for the sessions. For recruitment, phone and email were used. To check expertise, researchers’ own networks were used for the recruitment of experts. Meaning that an acquaintance from their networks could advise and endorse expertise of a potential expert. As a result of the researchers clinical and research background their network covered all profiles and representation of most city districts as well as relevant healthcare organizations. Eleven possible experts were approached, nine were included. One selected expert was excluded due to unavailability, the other due to lack of response. Expert saturation was confirmed by use of the DOLCE VITA research group’s opinion on the recruited experts’ ability to achieve to research aim. Enrollment of experts was completed in March 2021. Each expert signed an informed consent form before the first session. Figure 2 shows an overview of services in primary care, that are in operation in the Netherlands to prevent ED visits.

**Table 1:** Experts’ profiles

Profession	Type of care	Setting	Specific professional skills	Experts’ relevant additional skills
Data analyst health insurer	All types	Regional	Has knowledge on the data of large groups of older persons Has knowledge of the (financial) organization of care systems and regional differences	
District nurse	Chronic and temporary	Home	Has knowledge of early signs of developing acute care demands Has insight into functional, social and psychological aspects of community-dwelling older persons	Master student evidence-based practice
Emergency physician	Acute	Hospital: ED	Has knowledge on the spectrum of all ED patients and their acute care demands	
General practitioner (trained in elderly care)	Chronic and (sub)acute	Home Nursing home	Has insight into functional, social and psychological aspects of community-dwelling older persons as well as their developing acute care demands	
Geriatrician	(Sub)acute	Hospital: ED, ward, outpatient clinic	Has expertise in geriatric health conditions	Previous work experience as general practitioner and elderly care physician
Elderly care physician	Chronic and temporary	Home Nursing home	Has expertise in geriatric health conditions Has retrospective insight into the trajectory of acute care demands	
Nurse specialist geriatrics	(Sub)acute	Hospital: ED, ward, outpatient clinic	Has insight into functional, social and psychological aspects of older persons that visit the hospital	Previous work experience as ED nurse
Nurse transfer coordinator	(Sub)acute	Hospital: ED, ward	Has knowledge of organization of care and which type of care is appropriate	Previous work experience as nurse geriatrics Head of transfer department
Patient representative	All types	Local	Has knowledge of the perspective of older persons	Previous work experience as advisory board member for multiple elderly initiatives in Amsterdam

### Data collection, analyses, and model validation

Data were collected by video recording the sessions and capturing expert discussion on the online whiteboard, which IB marked directly during the sessions. Video records were transcribed verbatim, anonymized, and checked for accuracy. Data were analyzed and validated as part of the scripts and between sessions. In the scripts researcher OS and IB discussed all output, especially answers of experts that were clarified by the researchers. This was done plenary in iterative fashion throughout the GMB process with the expert group, to visualize their shared view in the most optimal way, as in line with Scriptapedia guidelines [22]. Between sessions researcher OS and IB completed visualization of session output and clarified expert answers, performed a search for supporting literature for each identified causal link and tested visualized interactions with validation criteria known as adjusted Goldratt’s categories of legitimate reservation [28]. In addition to literature, these criteria help to assess the clarity and acceptability of cause-and-effect formulations. Explanation of these criteria and the scores for the cause-and-effect formulations of this study’s CLD are provided in Appendix 4.

## Results

All sessions were held in April and May 2021 and resulted in a CLD that depicted the experts’ shared view on why people older than 65 years of age visit the ED in Amsterdam in the simplest way. All experts were present during session one, two, four and six. In session three the nurse transfer coordinator and the healthcare insurance data analyst were absent due to personal circumstances. Session five was a facultative voluntary session, in which the ED physician, geriatric nurse, and district nurse participated.

### A CLD of why people older than 65 years of age visit the ED in Amsterdam

**Fig. 3:**
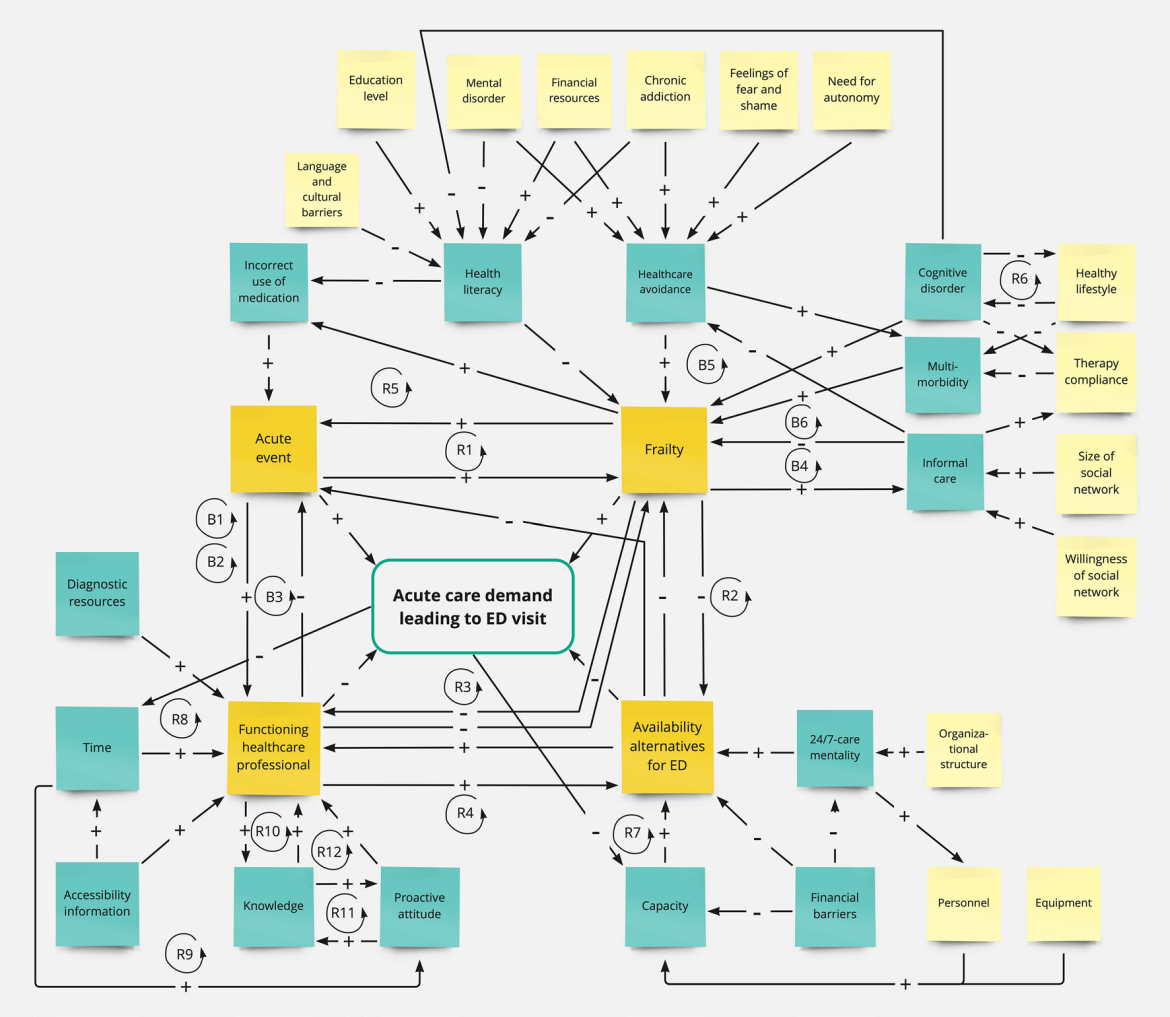
A CLD of why people older than 65 years of age visit the ED in Amsterdam. For the reader of this CLD, it has to be taken into account that factors have both qualitative as quantitative characteristics. For example, an increase in acute events could mean both the total number of acute events but also the severity of acute events. Together they represent a change in total amount of acute events at population level. Factors are placed in post its. The four direct factors are in dark yellow. In the view of the experts’, these factors have a direct causal effect on older persons’ ED visits. Blue factors are indirect underlying secondary factors. Light yellow factors are indirect underlying tertiary factors. A positive relationship, indicated by a + means that if A increases B will also increase. A negative relationship, indicated by a−means that if A increases B will decrease. A reinforcing loop has a spiraling effect on the original change (R), and can push a system out of balance, where a balancing loop (B) antagonizes the original effect and stabilizes the system. All factor definitions can be found in Appendix 2, their causal relations and supporting literature in Appendix 3

The CLD consisted of 4 direct factors, 29 underlying (secondary and tertiary) factors, 66 effects between the factors and 18 feedback loops. It is shown in Fig. 3. A list of all factor definitions can be found in Appendix 2. A list of all interactions and supporting literature can be found in Appendix 3. A validated list of all interactions can be found in Appendix 4.

### Direct factors, their interactions and feedback loops

Four factors were identified by the experts as direct factors leading to older persons’ ED visits. They included ‘acute event’, ‘frailty’, ‘functioning of the healthcare professional’ and ‘availability of alternatives for the ED’. All direct factors contributed to older persons’ ED visits, directly and indirectly by influencing each other. In addition, their effects were both amplified and balanced by their involvement in multiple feedback loops. In the following sections, first the direct factors and their effect on older persons’ ED visits are described, then their interactions and finally their feedback loops.

### Direct factors

#### Acute event

An ‘acute event’ was defined as a sudden and disruptive event at somatic, psychological, functional or social level. Its direct increasing effect on older persons’ ED visits was explained by the mechanism that these sudden and disruptive events at somatic, psychological, functional or social level generally result in an acute care demand that leads to an ED visit. Consensus was established quickly on the definition of this factor, because all experts saw it as intuitive. The examples given were mainly in the somatic and psychological domain, for example a delirium, a pneumonia or a fall. Excluded for the factor ‘acute event’ were gradually worsening chronic conditions, such as dementia or M. Parkinson, they were placed under the direct factor ‘frailty’. Only one secondary factor was found an important contributing factor for ‘acute event’, ‘incorrect use of medication’.

#### Frailty

‘Frailty’ was defined in this context as a state of reduced self-sustainability or increased dependence on others as a result of a complex interaction between somatic, psychological/cognitive, functional and social disabilities. Its direct increasing effect on older persons’ ED visits was explained by the mechanism that disequilibrium in these disabilities generally result in an acute care demand that leads to an ED visit. ‘Frailty’ as a factor was the most challenging for the experts’ group to define. This was due to the number of factors that underly ‘frailty’ as well as the polarity the name implies. Furthermore, they discussed whether underlying factors, such as chronic diseases (later specified as multi-morbidity), were truly underlying factors and not operating directly apart from the resultant ‘frailty’. As a result of in between session categorization and clarification, the researchers proposed the option to use frailty as a resultant factor. Experts agreed on using ‘frailty’ in this way. Six secondary factors were found important contributing factors for ‘frailty’, including health literacy, healthcare avoidance behavior, cognitive disorder, multi-morbidity and informal care. Moreover, eleven tertiary factors were identified, including ‘language/cultural barriers’, ‘education level’, ‘mental disorder’, ‘financial resources’, ‘chronic addiction’, ‘feelings of fear/shame’, ‘need for autonomy’, ‘healthy lifestyle’, ‘therapy compliance’, ‘size of social network’ and ‘willingness of social network’. To illustrate the interaction component of the factor ‘frailty’, healthcare avoidance behavior (secondary factor), for example, can be a result of an anxiety disorder (tertiary factor), which results in more ‘frailty’. When a strong informal care system (secondary factor) is present, such as an involved family, further deterioration of frailty can be prevented.

#### Functioning of the healthcare professional

‘Functioning of the healthcare professional’ was defined as the ability of healthcare professionals to recognize and acknowledge health problems in older persons, frailty in particular, as well as the ability to coordinate care between care providers to meet the patient’s needs. Its direct decreasing effect on older persons’ ED visits was explained by the mechanism that if healthcare professionals signal and coordinate care demands well, they generally prevent acute care demands that lead to ED visits. Consensus on this factor was established after several iterations on clarifying the signaling function. At first, it was described as “not being in the picture”, referring to older persons, frail in particular, not being in the picture for healthcare professionals. Yet there were three different components of this description including the absence of adequate signaling in the healthcare professional, the informal care provider and the patient. These three different components were placed in the factors; ‘functioning of the healthcare professional’, ‘informal care’ and ‘health literacy’. Coordination of care was clear to the experts. Finally, the signaling and coordinating function were merged to ‘functioning of the healthcare professional’ because it simplified the CLD considerably. Five secondary factors were found important contributing factors for ‘functioning of the healthcare professional’ including ‘diagnostic resources’, ‘accessibility of information’, ‘proactive attitude’, ‘knowledge’ and ‘time’.

#### Availability of alternatives for the ED

‘Availability of alternatives for the ED’ was defined as, the timely availability and accessibility of appropriate care options for patient’s needs, acute and chronic, the ED excluded. Its direct decreasing effect on older persons’ ED visits was explained by the mechanism that this timely availability generally prevents an acute care demand that leads to an ED visit. This factor was established after several clarifying iterations of the factor’lack of alternatives for the ED’. Experts established the importance of’lack of alternatives for the ED’ quickly but missed the timely component which in their opinion embodies the primary focus of this factor. Three secondary factors were found important contributing factors for ‘Availability of alternatives for the ED’ including ‘24/7 care mentality’, ‘capacity’ and ‘financial barriers. Three tertiary factors were identified, including ‘personnel’, ‘equipment’ and ‘organizational structure’. One expert depicts the situation of acute needs:

D6: “Needed care often isn’t available right away, often only tomorrow, or within 48 hours, when it really has to be accessible right away. In that case, I think the referrer has no other choice than to send a patient to the emergency room, which is also because there is often still an open end to what triggers the care problem.” Elderly care physician

Another example of the importance of timely availability given by the experts was the development of older persons acute care demands whilst waiting in a queue for a long-term care admittance in a nursing home. Due to unavailability of appropriate care ED visits increase.

### Factor interactions

A rise of ‘acute event’ had a direct increasing effect on older persons’ ED visits, but also an increasing effect on ‘frailty’ and on ‘functioning of the healthcare professional’. Therefore, in addition ‘acute event’ influenced older persons’ ED visits indirectly. The effect of ‘acute event’ on ‘frailty’ was explained by the mechanism that an ‘acute event’ generally pushes ‘frailty’ in to further reduced self-sustainability. For example, a pneumonia generally effects the physical function component of ‘frailty’ negatively and thereby decreases self-sustainability. An expert described the effect of ‘acute event’ on ‘functioning of the healthcare professional’ as follows:

D3: “An acute event is fairly prominent. No matter what kind of patient it is about, it always alarms, is prominently reported and triggers healthcare professionals.” Nurse specialist in geriatrics

A rise of ‘frailty’ had a direct increasing effect on older persons’ ED visits, but also an increasing effect on ‘acute event’ and a decreasing effect on ‘availability of alternatives for the ED’ as well as a decreasing effect on ‘functioning of the healthcare professional’. Therefore, in addition ‘frailty’ influenced older persons’ ED visits indirectly. The effect of ‘frailty’ on ‘acute event’ was explained by the mechanism that frail older persons have a considerably higher chance of developing an acute event due to their increased risk profile. For example, a chronic disease that predisposes for acute exacerbations. The effect on ‘availability of alternatives for the ED’ was due to the increased consumption of care capacity needed for frail older persons. The effect on ‘functioning of the healthcare professional’ was a result of the time-consuming effect, an increase of ‘frailty’, would have. Furthermore, to the opinion of the experts, ‘frailty’ itself did not have an increasing effect on the signaling abilities of the healthcare professional, because this would require schooling on ‘frailty’.

D1: “Knowledge of geriatric problems among general practitioners is variable. There is discussion in the field to include an elderly care physician on the GP-OOH services during shifts to manage geriatric problems” General practitioner

A rise of ‘functioning of the healthcare professional’ had a direct decreasing effect on older persons’ ED visits, but also a decreasing effect on ‘acute events’ and ‘frailty’, as well as an increasing effect on ‘availability of alternatives for the ED’. Therefore, in addition ‘functioning of the healthcare professional’ influenced older persons’ ED visits indirectly. The effect of ‘functioning of the healthcare professional’ on ‘acute event’ was explained by the mechanism that good signaling and coordination of the healthcare professional prevents further adverse effects of ‘acute event’ and therefore, led to a decrease of ‘acute event.’ An expert described the effect of ‘functioning of the healthcare professional’ on ‘frailty’ as follows:

D7: “With regard to frailty, what I often see in the emergency room is that people have already been frail for a long time. At some point the GP becomes aware of this frail state, yet appropriate care is often not organized in time. Then they present themselves for example, with a fall.” ED physician

The effect on ‘availability of alternatives for the ED’ was described by one expert as follows:

D5: “Better coordination by the health care professional ensures better use and accessibility of alternatives.” Transfer nurse

A rise of ‘availability of alternatives for the ED’ had a direct decreasing effect on older persons’ ED visits, but also a decreasing effect on ‘acute event’ and ‘frailty’ as well as an increasing effect on ‘functioning of the healthcare professional’. Therefore, in addition ‘availability of alternatives for the ED’ influenced older persons’ ED visits indirectly. The effects of ‘availability of alternatives for the ED’ on the other direct factors were explained by the following mechanisms. When appropriate needed care is timely available for both, patient, informal care giver and healthcare professional to organize, acute events can often be prevented in a subacute state, frailty can be managed appropriately and healthcare professionals can perform better in their function.

### Feedback loops

**Fig. 4:**
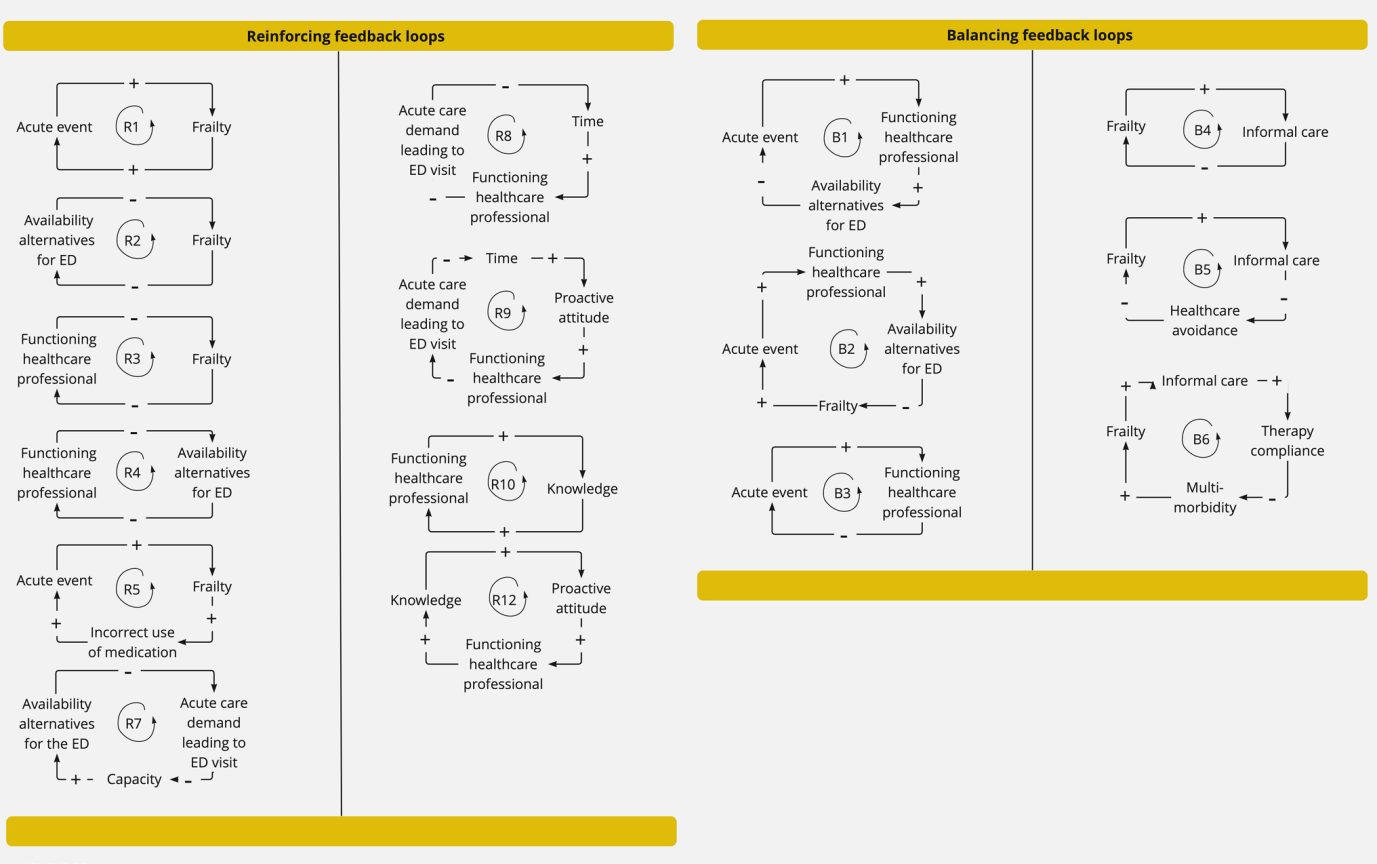
Direct factors involving feedback loops

‘Acute event’ was involved in five feedback loops as a result of the causal relations perceived by the experts that were visualized in the CLD. For example, an increase in either ‘acute event’ or ‘frailty’ would result in an increase of the other, thereby creating a reinforcing feedback loop contributing to ED visits (R1). All feedback loops involving direct factors are shown in Fig. 4. A list of all feedback loops can be found in Appendix 5.

‘Frailty’ was involved in eight feedback loops. Because of ‘frailty’s involvement in the feedback loops R3 and R2 an increase of the amount of frail older persons would also have a spiraling deteriorating effect on ‘functioning of the healthcare professional’ as well as ‘availability of alternatives for the ED’. Thereby resulting in an increase of older persons’ ED visits in multiple ways. On the other hand, ‘frailty’ and its effect on the system can be balanced by good informal care. The effect of good informal care balances ‘frailty’ via several feedback loops including B4, B5 and B6.

‘Healthcare professional functioning’ was involved in nine feedback loops. Because of ‘Healthcare professional functioning’ involvement in B3 ‘acute event’ was balanced with good signaling and coordination of care. Furthermore, ‘Healthcare professional functioning’ can be boosted by providing more time and schooling focused on geriatric medicine, thereby using the spiraling effect of R8, R9, R10, R11, and R12.

‘Availability of alternatives for the ED’ was involved in five feedback loops. Because of ‘Availability of alternatives for the ED’s involvement in the reinforcing feedback loops R4 and R7 an improvement in availability of appropriate care options for older persons would not only have a direct spiraling effect lowering older persons’ ED visits but also via boosting functioning of the health care professional. The feedback loops illustrate the interconnectedness of the direct factors and the complex effects they have on older persons’ ED visits as well as each other.

## Discussion

In this GMB study, a CLD was formed, describing the etiology of older persons’ ED visits in the Amsterdam region from the shared view of an expert group, trying to capture this complex problem in its simplest way. We identified four direct factors, 29 indirect factors, 66 interactions and 18 feedback loops. This result shows the highly complex system of interacting factors that drive older adults to emergency visits. In line with previous studies, we report on extensive interaction, between both patient, healthcare professional and healthcare organization based contributing factors and thereby highlight their importance [4, 8]. The extensiveness of the CLD could be due to older patients’ characteristics. For example, atypical disease presentation of frail older persons is often the result of multicausal health problems [29, 30]. Moreover, this could be due to the organization of the healthcare system since medicine focuses on single diseases [12]. Often healthcare system organization does not match older persons’ characteristics and multiple care needs. Therefore, it could contribute to ED visits. Furthermore, the coherence captured in the CLD’s feedback loops identifies options for potentially effective interventions. For instance, the reinforcing effect of the feedback loops involving ‘knowledge’ and ‘time’, can be used to boost ‘healthcare professional functioning’ [31].

### Insights in key factors, interactions and feedback loops

#### Acute events

In our study, ‘acute event’ was identified as a key contributing factor for older persons’ ED visits. Previous studies show that ‘acute events’, such as fall related injuries (15–30%), dyspnea and chest pain (15–20%), infections (5–15%), adverse drug events (about 10%) and delirium (7–10%) cause a large number of older adults’ ED visits [31–34]. An observational study found that 9% of older patients in a Swiss ED were sent in for the inability to take care of themselves [29]. Interestingly, 51% of these “social cases” appeared to have an underlying acute medical problem that needed immediate treatment. These results are in line with the experts’ opinion that acute events enhance frailty and result in ED visits via that pathway. Since acute events lead to more frailty and frailty to more acute events a feedback loop is present.

#### Frailty

Frailty was identified as a key contributing factor, defined as a result of specific underlying factors. Literature reports extensively on the relationship between older persons’ ED visits and the underlying factors for ‘frailty’ identified in this study. Multimorbidity, polypharmacy and cognitive impairment are associated with an increased risk for ED visits and revisits [35–37]. Various studies state that healthcare avoidance contributes to older adults’ ED visits [38–40]. Lutz et al. identified that patients’ financial resources, substance abuse, loneliness and desire to maintain their independence drive them to seek non-urgent care in the ED [38]. These factors are depicted in the CLD as underlying causes of healthcare avoidance. In regard to informal care, a recent systematic review showed that older persons with weak social relationships are at higher risk for hospital readmissions and longer hospital stays [41]. However, little evidence was found for an association with ED visits. This inconsistency is illustrated in the CLD. Herein informal care has no direct impact on lowering ED visits, but positively influences the system indirectly in multiple ways, such as by reducing frailty and enhancing therapy adherence. Other studies have identified health literacy as a contributing factor [42, 43]. Especially, the ability of older persons to interpret their symptoms and to navigate within the healthcare system seem to be significant contributors to ED visits [4, 6, 38]. Finally, our studies’ CLD provides a possible explanation why some of these factors have not shown an association with ED visits, that could be because they effect ED visits indirectly.

#### Healthcare professional functioning

In this study, the experts argued that ED visits increase when healthcare professionals fail to signal their patients’ care needs (both ‘acute event’ and ‘frailty’ driven) and coordinate care accordingly. This idea is supported by a several studies [6, 8, 39]. An enquiry among 1600 general practitioners (GPs) in the Netherlands reported that, the amount of older persons seeking care, their complexity, the healthcare system organization and government policy to let people live at home as long as possible, contribute significantly to time shortage [44]. The time shortage itself leads to lower quality of care, through insufficient time to help the patient appropriately as well as falling behind on education. This enquiry illustrates the effects of frailty on the functioning of the GP and it can have on ED visits. In addition, Dutch emergency physicians report that they feel insufficiently educated to treat older persons [45]. These reports endorse the feedback loops depicted in this CLD involving the health care professional.

#### Availability of ED alternatives

Finally, the availability of alternative care options outside the ED was identified as a significant direct causal factor. This is in line with several studies [6, 46]. In an interview study, GPs and ED personnel pointed out that a shortage of homecare personnel, reduction in nursing home beds, inability of the healthcare system to have short-term follow-up diagnostics, and waiting times for out-patient-clinics contribute to ED return visits in the Netherlands [6]. Likewise, a mixed-method study showed that a lack of availability and accessibility of GPs, GP out-of-hours services, at-home multidisciplinary teams and community beds explained variance in ED visits between different regions in England [4]. These studies endorse the role availability of ED alternatives plays in interaction with health care professionals functioning and frailty, contributing both directly as indirectly to ED visits.

A strength of this study is the choice of model. In contrast to other models, this CLD gives an instant overview of a problems’ key mechanisms, therefore, it can be used as a tool for the work floor, research and education to help explain a problems’ etiology. An additional strength is that the CLD was built via an interdisciplinary GMB approach and therefore, depicted the shared view of various experts in the simplest way. At last, this GMB approach included an experienced older patient’s representative and therefore provided the opportunity to test suggested factors and effects immediately on older persons perspective.

A possible limitation of this study is that a CLD in definition is a qualitative model. It is, therefore, to some extent unique to the group and problem. Third, we aimed to capture only the most important contributing factors and interactions for the total population of older persons in Amsterdam. Consequently, this CLD does not include all factors at play and in-depth insight in subpopulations. This could mean that for some subpopulations important factors at play are not captured in this CLD. Fourth, by aiming to develop a CLD to depict the studied problems’ etiology in 2019 pre-COVID, while conducting the study during COVID in 2021, the experts’ view on older persons ED visits might have subconsciously been influenced by their experience of contributing factors during this pandemic. Lastly, one could argue if our expert group included all essential stakeholders.

To further enhance the understanding of contributing factors to older persons’ ED visits and their interactions in the future, research should be focused on repeating comparable GMB studies in other areas, both similar in healthcare organization and population, as well as less similar, often rural, areas. Furthermore, in the experts’ view health literacy and health care avoidance behavior specifically, play an important role in the etiology of frailty that leads to older persons’ ED visits. Therefore, future GMB studies should aim to capture what drives the etiology of these risk factors in focused CLD’s to develop interventions that can prevent or reduce their risk. Moreover, extra representatives for these subpopulation and their informal care givers as well as experts from more sociological disciplines should then be included to develop in-depth insight. Lastly, the feedback loops that were identified in this study’s CLD, such as R8–R12 (healthcare professional functioning can be boosted by providing more time and schooling focused on geriatric medicine), should be investigated for their potential as effective interventions for older persons ED visit by testing them through system dynamics simulation models.

## Conclusion

This study shows that the etiology of older persons’ ED visits in Amsterdam entails a highly complex system of interacting factors that drive older persons to an emergency department. This system pivots around four key contributing factors including: functioning of the healthcare professional, availability of alternatives for the ED, acute events and frailty. All contributed directly and indirectly by interacting with each other as well as their many underlying factors. Visualized in the CLD formed in this study, the role interactions of contributing factors can play in the etiology of older persons’ ED visits is elucidated and thereby helps to better understand them. This CLD can help to explore effective interventions for the increasing numbers of older adults in the ED.

### Background information on researchers

OS is an MD PhD student on the DOLCE VITA project, researching the etiology of older adults’ ED visits, and a fifth-year internal medicine resident specializing in geriatric medicine. He has extensive experience in clinically assessing older adults at the ED in Amsterdam, both at a city-center hospital and the academic center. Furthermore, he has extensive experience in moderating group discussion and has conducted focus groups in the past.

IB is a medical student in her last year of training and is writing her master’s thesis on the case study under supervision of OS, HW and BB. She has experience in clinically assessing older adults at the ED of a city-center hospital in Amsterdam and moderating group discussion.

HW is an MD PhD internist-geriatrician and is head of the department of geriatric medicine at Amsterdam UMC. She has extensive expertise in geriatric medicine and in conducting focus groups. She is a co-supervisor of OS.

ER is professor at the Methodology Department of the Nijmegen School of Management, Radboud University (the Netherlands). He is specialized in GMB methodology.

BB is professor at the department of geriatric medicine at Amsterdam UMC and is chair of the Dutch society for Nurses. She is specialized in Acute geriatric care. She has extensive expertise in the acute geriatric research field. She is a supervisor of OS.

## Supplementary Information

Below is the link to the electronic supplementary material.

Supplementary file1 (DOCX 856 KB)

## Data Availability

The majority of data generated and analyzed during the current study are publicly available and included in either the manuscript or additional file. Restrictions apply to the availability of the transcripts, which were used under approval of the experts for the current study, and so are not publicly available. Transcripts are however available from the authors upon reasonable request and with the permission of the experts. Transcripts are in Dutch.

## References

[1] Ukkonen M, Jämsen E, Zeitlin R (2019). Emergency department visits in older patients: a population-based survey. BMC Emerg Med.

[2] Cylus J, Figueras J, Normand C (2019) The economics of healthy and active ageing series, will population ageing spell the end of the welfare state? A review of evidence and policy options. European Observatory on Health Systems and Policies. European Observatory Policy Briefs., Copenhagen, Denmark 31820887

[3] Van BB (2018). Symptoombestrijding Naar Duurzame Acute Ouderenzorg (Dutch).

[4] O’Cathain A, Knowles E, Turner J (2014). Explaining variation in emergency admissions: a mixed-methods study of emergency and urgent care systems. Health Serv Deliv Res.

[5] Dufour I, Chouinard MC, Dubuc N (2019). Factors associated with frequent use of emergency-department services in a geriatric population: a systematic review. BMC Geriatr.

[6] Driesen BEJM, Merten H, Wagner C (2020). Unplanned return presentations of older patients to the emergency department: a root cause analysis. BMC Geriatr.

[7] Fluitman KS, van Galen LS, Merten H (2016). Exploring the preventable causes of unplanned readmissions using root cause analysis: coordination of care is the weakest link. Eur J Intern Med.

[8] Kolk D, Kruiswijk AF, MacNeil-Vroomen JL (2021). Older patients’ perspectives on factors contributing to frequent visits to the emergency department: a qualitative interview study. BMC Public Health.

[9] Hoitinga G, Kolk D, Rijkerberg S *et al.* Perceptions of the frail elderly on contributing factors causing the onset of crises; a qualitative study. Thesis. Amsterdam UMC location University of Amsterdam, Internal Medicine, section of Geriatric Medicine. 2021.

[10] Uleman JF, Melis RJF, Quax R (2021). Mapping the multicausality of Alzheimer’s disease through group model building. Geroscience.

[11] Inouye SK, Studenski S, Tinetti ME (2007). Geriatric syndromes: clinical, research and policy implications of a core geriatric concept. J Am Geriatr Soc.

[12] Olde Rikkert MGM, Melis RJF, Cohen AA (2022). Why illness is more important than disease in old age. Age Ageing.

[13] Verhaegh KJ, MacNeil-Vroomen JL, Eslami S (2014). Transitional care interventions prevent hospital readmissions for adults with chronic illnesses. Health Aff.

[14] Suijker JJ, van Rijn M, Buurman BM (2016). Effects of nurse-led multifactorial care to prevent disability in community-living older people: cluster randomized trial. PLoS One.

[15] Bleijenberg N, Drubbel I, Schuurmans MJ (2016). Effectiveness of a proactive primary care program on preserving daily functioning of older people: a cluster randomized controlled trial. J Am Geriatr Soc.

[16] Preston L, Conroy SP, Ablard S (2021). Improving outcomes for older people in the emergency department: a review of reviews. Emerg Med J.

[17] Sterman J (2000). Business dynamics : systems thinking and modelling for a complex world.

[18] Aronson D, Angelakis D. Step-by-step stocks and flows: improving the rigor of your thinking. https://thesystemsthinker.com/step-by-step-stocks-and-flows-improving-the-rigor-of-your-thinking/. Accessed 1 June 2022

[19] Lane DC, Sterman JD (2011). Jay Wright Forrester. International series in operations research and management science.

[20] Andersen DF, Richardson GP (1997). Scripts for group model building. Syst Dyn Rev.

[21] Hovmand PS, Profile S, Rouwette EAJA et al (2011) Scriptapedia: a handbook of scripts for developing structured group model building. System Dynamics Conference., Washington D.C., pp 1476–1491

[22] Scriptapedia. https://en.wikibooks.org/wiki/Scriptapedia.

[23] Smeekes O, Willems H, Blomberg I et al (2023) Implementing online group model building to unravel complex geriatric problems: a methodological description. BMC Geriatrics (in press) 10.1186/s12877-023-04110-x PMC10339532 37438723

[24] Richardson GP (1991). Feedback thought in social science and systems theory.

[25] Vermaak H (2016). Using causal loop diagrams to deal with complex issues: mastering an instrument for systematic and interactive change. Consultation for organizational change revisited.

[26] What is System Dynamics. https://systemdynamics.org/what-is-system-dynamics/.

[27] Wilkerson B, Aguiar A, Gkini C (2020). Reflections on adapting group model building scripts into online workshops. Syst Dyn Rev.

[28] Burns JR, Musa P. Structural Validation of Causal Loop Diagrams. In: Atlanta SD Conference. 2001, 1–13.

[29] Rutschmann O, Chevalley T, Zumwald C (2005). Pitfalls in the emergency department triage of frail elderly patients without specific complaints. Swiss Med Wkly.

[30] Rockwood K, Howlett SE (2018). Fifteen years of progress in understanding frailty and health in aging. BMC Med.

[31] Samaras N, Chevalley T, Samaras D (2010). Older patients in the emergency department: a review. Ann Emerg Med.

[32] Aminzadeh F, Dalziel WB (2002). Older adults in the emergency department: a systematic review of patterns of use, adverse outcomes, and effectiveness of interventions. Ann Emerg Med.

[33] Schrijver E, Toppinga Q, de Vries O (2013). An observational cohort study on geriatric patient profile in an emergency department in the Netherlands. Neth J Med.

[34] Hohl CM, Dankoff J, Colacone A (2001). Polypharmacy, adverse drug-related events, and potential adverse drug interactions in elderly patients presenting to an emergency department. Ann Emerg Med.

[35] de Gelder J, Lucke JA, de Groot B (2018). Predictors and outcomes of revisits in older adults discharged from the emergency department. J Am Geriatr Soc.

[36] Dufour I, Chiu Y, Courteau J (2020). Frequent emergency department use by older adults with ambulatory care sensitive conditions: a population-based cohort study. Geriatr Gerontol Int.

[37] Chamberlain AM, Rutten LJF, Jacobson DJ (2019). Multimorbidity, functional limitations, and outcomes: Interactions in a population-based cohort of older adults. J Comorb.

[38] Lutz BJ, Hall AG, Vanhille SB (2018). A framework illustrating care-seeking among older adults in a hospital emergency department. Gerontologist.

[39] Verhaegh M, Snijders F, Janssen L (2019). Perspectives on the preventability of emergency department visits by older patients. Neth J Med.

[40] Ionescu-Ittu R, McCusker J, Ciampi A (2007). Continuity of primary care and emergency department utilization among elderly people. CMAJ.

[41] Valtorta NK, Moore DC, Barron L (2018). Older adults’ social relationships and health care utilization: a systematic review. Am J Public Health.

[42] Balakrishnan MP, Herndon JB, Zhang J (2017). The association of health literacy with preventable ED visits: a cross-sectional study. Acad Emerg Med.

[43] Greene JC, Haun JN, French DD (2019). Reduced hospitalizations, emergency room visits, and costs associated with a web-based health literacy, aligned-incentive Intervention: mixed methods study. J Med Internet Res.

[44] Newcom Research & Consultancy (2018) Werkdruk huisarts bedreigt kwaliteit zorg. (Dutch) https://www.newcom.nl/2018-werkdruk-huisarts/. Accessed 1 June 2022.

[45] Sir Ö, Hesselink G, Schoon Y (2021). Dutch emergency physicians insufficiently educated in geriatric emergency medicine: results of a nationwide survey. Age Ageing.

[46] van den Broek S, Heiwegen N, Verhofstad M (2020). Preventable emergency admissions of older adults: an observational mixed-method study of rates, associative factors and underlying causes in two Dutch hospitals. BMJ Open.

[47] Uw onderzoek: WMO-plichtig of niet? | Onderzoekers | Centrale Commissie Mensgebonden Onderzoek

[48] WMA Declaration of Helsinki–Ethical Principles for Medical Research Involving Human Subjects–WMA–The World Medical Association 19886379

