# Supplementary material for: A causal loop diagram of older persons’ emergency department visits and interactions of its contributing factors: a group model building approach
Source: Eur Geriatr Med. 2023 Jun 30;14(4):837–49. doi: 10.1007/s41999-023-00816-8 (PMC10447269; doi:10.1007/s41999-023-00816-8)
Supplement: Supplementary file 1 — Supplementary file1 (DOCX 856 KB) [file 41999_2023_816_MOESM1_ESM.docx]

**A causal loop diagram of older persons’ emergency department visits and interactions of its contributing factors: a group model building approach**

**Supplementary** **data**

1. Definition of the Amsterdam area and setting
2. List of factor definitions
3. Table of all interactions and supporting literature
4. Validation table of interactions: Goldratt’s criteria for legitimate reservation
5. Overview of all feedback loops
6. **Definition of the Amsterdam area and setting**

This research was nested within the Data driven Optimization for a Vital elderly care system in the Netherlands (DOLCE VITA) project. The project aims to elucidate acute care for older adults in the Netherlands, particularly in Amsterdam, and to improve future policy-making using model development. The program involved two PhDs with a medical and mathematical background, and multiple clinicians and researchers from medical and mathematical fields. In the DOLCE VITA project, Amsterdam included Amstelveen and Diemen and this geographical area has nearly one million inhabitants with a diverse demography and a range of healthcare providers, including one tertiary hospital (two locations), two larger city hospitals (one with two locations), and one smaller teaching hospital. This GMB study was conducted in the same geographical region as the DOLCE VITA research project.

1. **List of factor definitions**

**24/7-care mentality**

Healthcare service guidelines that determine opening hours for contact and care, including examinations, admissions or referrals.

**Accessibility of information**

The degree in which healthcare professionals can access relevant patient related information recorded by other healthcare professionals at the time of providing care, including medical history, medication use and treatment wishes.

**Acute care demand leading to an ED visit**

All ED visits of older persons older than 65 years of age in Amsterdam.

**Acute event**

A sudden and disruptive event at somatic, psychological, functional or social level.

**Availability of alternatives for ED**

The timely availability and accessibility of appropriate care options for patient’s needs, acute and chronic, the ED excluded.

**Capacity**

The total amount of patients that can receive care within a healthcare service per amount of time.

**Care avoidance behavior**

Patient’s behavior that results in avoidance of care or support by others, due to either physical illness, mental illness or unwillingness of the person to seek help.

**Chronic addiction**

Compulsory substance use.

**Cognitive disorder**

Impaired cognitive functioning due to dementia, mild cognitive impairment or mental disability.

**Diagnostic resources**

Diagnostic equipment, tools and methodology that is required to adequately diagnose illness.

**Education level**

The level of education an older person has.

**Equipment**

Materials that are needed to provide care by personnel, for example, beds.

**Feelings of fear and shame**

Fear or shame for (potential) illness or personal situation.

**Financial barriers**

Financial regulations set by the government, healthcare insurers or care-providing services that obstruct delivery of appropriate care for patients.

**Financial resources**

A persons’ total amount of potentially accessible money.

**Frailty**

A state of reduced self-sustainability and increased dependence on others as a result of a complex interaction between somatic, psychological/cognitive, functional and social disabilities

**Functioning of healthcare professionals**

The ability of healthcare professionals to recognize and acknowledge health problems in older persons, frailty in particular, as well as the ability to coordinate care between care providers to meet the patient’s needs.

**Health literacy**

A person’s ability to gain access to, understand and use information in ways that promote and maintain good health.

**Healthy lifestyle**

A lifestyle that lowers the risk of developing illness.

**Incorrect use of medication**

Not taking medication as prescribed.

**Informal care**

Non-professional support from the person’s immediate social circle.

**Knowledge**

Healthcare professional’s knowledge on medical topics (e.g. older adults’ characteristics, disease presentations or treatment options) and organizational topics (e.g. how to navigate within the local healthcare system).

**Language and cultural barriers**

Incomprehension of communication with the healthcare system by older persons as a result of different language or cultural background.

**Mental disorder**

Psychiatric disorders, excluding addiction, dementia, mild cognitive impairment or mental disability.

**Multimorbidity**

Patients with two or more chronic health conditions that persist life-long, occasionally exacerbate and typically progress over time.

**Need for autonomy**

The wish to be self-dependent.

**Organizational structure**

The way in which a care-providing service is designed and governed, for example which activities and services it can provide and who is in charge of decision making.

**Personnel**

All healthcare personnel essential for providing care directly and indirectly.

**Proactive attitude**

Healthcare professionals acting in anticipation of future problems, needs or changes of their patients, rather than reacting to problems when they have already emerged, as well as taking responsibility for delivering good quality care.

**Size of social network**

Number of acquaintances.

**Willingness of social network**

A social networks willingness to provide informal care.

**Therapy compliance**

Following treatment as advised.

**Time**

The amount of time available for healthcare professionals to spend on delivering good quality care for their patients

1. **Table of all interactions supporting literature**

| Nr. | From | Causal relationship | To |  |
| --- | --- | --- | --- | --- |
| *1* | Acute event | + | Acute care demand leading to ED visit | [1–4] |
| *2* | Acute event | + | Frailty | [5,6] |
| *3* | Acute event | + | Functioning healthcare professional | [7–9] |
| *4* | Frailty | + | Acute care demand leading to ED visit | [5,6] |
| *5* | Frailty | + | Acute event | [5,6] |
| *6* | Frailty | - | Availability alternatives for ED | [6,10,11] |
| *7* | Frailty | - | Functioning healthcare professional | [6,10,11] |
| *8* | Frailty | + | Informal care | [12,13] |
| *9* | Frailty | + | Incorrect use of medication | [1–4] |
| *10* | Availability alternatives for ED | - | Acute care demand leading to ED visit | [6,11,14] |
| *11* | Availability alternatives for ED | - | Acute event | [6,11] |
| *12* | Availability alternatives for ED | - | Frailty | [6,11] |
| *13* | Availability alternatives for ED | + | Functioning healthcare professional | [11,15] |
| *14* | Functioning healthcare professional | - | Acute care demand leading to ED visit | [11,16,17] |
| *15* | Functioning healthcare professional | - | Acute event | [11,16,17] |
| *16* | Functioning healthcare professional | - | Frailty | [10,11,15–17] |
| *17* | Functioning healthcare professional | + | Availability alternatives for ED | [11,14,15] |
| *18* | Functioning healthcare professional | + | Knowledge | [15,18] |
| *19* | Acute care demand leading to ED visit | - | Time | [11,15] |
| *20* | Acute care demand leading to ED visit | - | Capacity | [11,15] |
| *21* | Incorrect use of medication | + | Acute event | [1–4] |
| *22* | Health literacy | + | Frailty | [6,10,11,19,20] |
| *23* | Health literacy | - | Incorrect medication use | [10] |
| *24* | Healthcare avoidance | + | Frailty | [10,16,21] |
| *25* | Healthcare avoidance | + | Multi-morbidity | [6] |
| *26* | Cognitive disorder | + | Frailty | [6,10,11,22–24] |
| *27* | Cognitive disorder | - | Health literacy | [25] |
| *28* | Cognitive disorder | - | Healthy lifestyle | [26] |
| *29* | Cognitive disorder | - | Therapy compliance | [27] |
| *30* | Multimorbidity | + | Frailty | [3,6,22–24] |
| *31* | Informal care | - | Frailty | [28,29] |
| *32* | Informal care | - | Healthcare avoidance | [10,29] |
| *33* | Informal care | + | Therapy compliance | [10] |
| *34* | Financial barriers | - | Availability alternatives for ED | [6,11] |
| *35* | Financial barriers | - | Capacity | [6,11] |
| *36* | Financial barriers | - | 24/7-care mentality | [6,11] |
| *37* | 24/7-care mentality | + | Availability alternatives for ED | [6,11] |
| *38* | 24/7-care mentality | + | Personnel | [6,11] |
| *39* | Capacity | + | Availability alternatives for ED | [6,11] |
| *40* | Diagnostic resources | + | Functioning healthcare professional | [11] |
| *41* | Time | + | Functioning healthcare professional | [15] |
| *42* | Time | + | Proactive attitude | [15] |
| *43* | Accessibility of information | + | Functioning healthcare professional | [30] |
| *44* | Accessibility of information | + | Time | [30] |
| *45* | Knowledge | + | Functioning healthcare professional | [18] |
| *46* | Knowledge | + | Proactive attitude | [18] |
| *47* | Proactive attitude | + | Functioning healthcare professional | [18] |
| *48* | Proactive attitude | + | Knowledge | [18] |
| *49* | Language and cultural barriers | - | Health literacy | [31] |
| *50* | Education level | + | Health literacy | [6] |
| *51* | Mental disorder | - | Health literacy | [32] |
| *52* | Mental disorder | + | Healthcare avoidance | [10] |
| *53* | Financial resources | + | Health literacy | [6,10,28] |
| *54* | Financial resources | + | Healthcare avoidance | [10] |
| *55* | Feelings of fear and shame | + | Healthcare avoidance | [10] |
| *56* | Chronic addiction | - | Health literacy | [10] |
| *57* | Chronic addiction | + | Healthcare avoidance | [10] |
| *58* | Need for autonomy | + | Healthcare avoidance | [10] |
| *59* | Healthy lifestyle | - | Cognitive disorder | [6,33] |
| *60* | Healthy lifestyle | - | Multimorbidity | [6] |
| *61* | Therapy compliance | - | Multimorbidity | [34,35] |
| *62* | Size of social network | + | Informal care | [10,29] |
| *63* | Willingness of social network | + | Informal care | [10,29] |
| *64* | Organizational structure | + | 24/7-care mentality | [6,11,15] |
| *65* | Personnel | + | Capacity | [6,11] |
| *66* | Equipment | + | Capacity | [6,11] |

1. **Validation table of interactions: Goldratt’s criteria for legitimate reservation**

We tested the validity of the causal relationships manually using the adjusted Goldratt validation criteria described by Burns et al. [36]. The criteria include: clarity, quantity existence, causality existence, cause insufficiency, additional cause, cause-effect reversal, predicted effect and tautology. In sum, Burns et al. describe testing for these criteria as follows: Questions that get addressed, during the clarity investigation of the CLD include: 1) is any additional verbal explanation required for the cause and its effect to be understood; 2) is the connection between cause and effect convincing at “face value;” 3) is this a “long link” (i.e., missing intermediate quantities and edges). Quantity existence involves hypothesizing if the factor can be translated to units. Causality existence questions the reality of the suggested causal link. Cause insufficiency questions, if the causal link, by itself, can create the effect that is expected in the target quantity. In additional cause assessment, it is questioned if the link is not unique and that other cause variables could independently produce the same effect. Cause-effect reversal is questioning if the stated effect is really the cause and conversely. The question asked in predicted effect existence is, if the suggested cause variable is really the culprit, what other effects could we also observe as a result of this hypothesized cause? And lastly, links are checked for cause effect tautology. Scoring was performed based on experts’ argumentation captured in the transcripts as well as on researchers’ application of logics. The numbers given to the causal relationships are equal to the numbers in ‘’the list of all interactions’’. “Zero/0” means that the validity criterium is demonstrated, “cross/X” means that the validity criterium is not or not completely demonstrated.

| Nr. | Clarity | Quantity  existence | Causality  existence | Cause  insufficiency | Additional  cause | Cause-effect  reversal | Predicted  effect | Tautology |
| --- | --- | --- | --- | --- | --- | --- | --- | --- |
| 1 | 0 | 0 | 0 | 0 | 0 | 0 | 0 | 0 |
| 2 | 0 | 0 | 0 | 0 | 0 | 0 | 0 | 0 |
| 3 | 0 | 0 | 0 | 0 | 0 | 0 | 0 | 0 |
| 4 | 0 | 0 | 0 | 0 | 0 | 0 | 0 | 0 |
| 5 | 0 | 0 | 0 | 0 | 0 | 0 | 0 | 0 |
| 6 | x | 0 | 0 | 0 | 0 | 0 | 0 | 0 |
| 7 | x | 0 | 0 | 0 | 0 | 0 | 0 | 0 |
| 8 | 0 | 0 | 0 | 0 | 0 | 0 | 0 | 0 |
| 9 | 0 | 0 | 0 | 0 | 0 | 0 | 0 | 0 |
| 10 | 0 | 0 | 0 | 0 | 0 | 0 | 0 | 0 |
| 11 | 0 | 0 | 0 | 0 | 0 | 0 | 0 | 0 |
| 12 | 0 | 0 | 0 | 0 | 0 | 0 | 0 | 0 |
| 13 | 0 | 0 | 0 | 0 | 0 | 0 | 0 | 0 |
| 14 | 0 | 0 | 0 | 0 | 0 | 0 | 0 | 0 |
| 15 | 0 | 0 | 0 | 0 | 0 | 0 | 0 | 0 |
| 16 | 0 | 0 | 0 | 0 | 0 | 0 | 0 | 0 |
| 17 | x | 0 | 0 | 0 | 0 | 0 | 0 | 0 |
| 18 | 0 | 0 | 0 | 0 | 0 | 0 | 0 | 0 |
| 19 | 0 | 0 | 0 | 0 | 0 | 0 | 0 | 0 |
| 20 | 0 | 0 | 0 | 0 | 0 | 0 | 0 | 0 |
| 21 | 0 | 0 | 0 | 0 | 0 | 0 | 0 | 0 |
| 22 | 0 | 0 | 0 | 0 | 0 | 0 | 0 | 0 |
| 23 | 0 | 0 | 0 | 0 | 0 | 0 | 0 | 0 |
| 24 | 0 | 0 | 0 | 0 | 0 | 0 | 0 | 0 |
| 25 | 0 | 0 | 0 | 0 | 0 | 0 | 0 | 0 |
| 26 | 0 | 0 | 0 | 0 | 0 | 0 | 0 | 0 |
| 27 | 0 | 0 | 0 | 0 | 0 | 0 | 0 | 0 |
| 28 | 0 | 0 | 0 | 0 | 0 | 0 | 0 | 0 |
| 29 | 0 | 0 | 0 | 0 | 0 | 0 | 0 | 0 |
| 30 | 0 | 0 | 0 | 0 | 0 | 0 | 0 | 0 |
| 31 | 0 | 0 | 0 | 0 | 0 | 0 | 0 | 0 |
| 32 | 0 | 0 | 0 | 0 | 0 | 0 | 0 | 0 |
| 33 | 0 | 0 | 0 | 0 | 0 | 0 | 0 | 0 |
| 34 | 0 | 0 | 0 | 0 | 0 | 0 | 0 | 0 |
| 35 | 0 | 0 | 0 | 0 | 0 | 0 | 0 | 0 |
| 36 | 0 | 0 | 0 | 0 | 0 | 0 | 0 | 0 |
| 37 | 0 | 0 | 0 | 0 | 0 | 0 | 0 | 0 |
| 38 | x | 0 | 0 | 0 | 0 | 0 | 0 | 0 |
| 39 | 0 | 0 | 0 | 0 | 0 | 0 | 0 | 0 |
| 40 | 0 | 0 | 0 | 0 | 0 | 0 | 0 | 0 |
| 41 | 0 | 0 | 0 | 0 | 0 | 0 | 0 | 0 |
| 42 | 0 | 0 | 0 | 0 | 0 | 0 | 0 | 0 |
| 43 | 0 | 0 | 0 | 0 | 0 | 0 | 0 | 0 |
| 44 | 0 | 0 | 0 | 0 | 0 | 0 | 0 | 0 |
| 45 | 0 | 0 | 0 | 0 | 0 | 0 | 0 | 0 |
| 46 | 0 | x | 0 | 0 | 0 | 0 | 0 | 0 |
| 47 | 0 | 0 | 0 | 0 | 0 | 0 | 0 | 0 |
| 48 | 0 | 0 | 0 | 0 | 0 | 0 | 0 | 0 |
| 49 | 0 | 0 | 0 | 0 | 0 | 0 | 0 | 0 |
| 50 | 0 | 0 | 0 | 0 | 0 | 0 | 0 | 0 |
| 51 | 0 | 0 | 0 | 0 | 0 | 0 | 0 | 0 |
| 52 | 0 | 0 | 0 | 0 | 0 | 0 | 0 | 0 |
| 53 | 0 | 0 | 0 | x | 0 | 0 | x | 0 |
| 54 | 0 | 0 | 0 | 0 | 0 | 0 | 0 | 0 |
| 55 | 0 | 0 | 0 | 0 | 0 | 0 | 0 | 0 |
| 56 | x | 0 | 0 | x | 0 | 0 | x | 0 |
| 57 | 0 | 0 | 0 | 0 | 0 | 0 | 0 | 0 |
| 58 | 0 | 0 | 0 | 0 | 0 | 0 | 0 | 0 |
| 59 | 0 | 0 | 0 | 0 | 0 | 0 | 0 | 0 |
| 60 | 0 | 0 | 0 | 0 | 0 | 0 | 0 | 0 |
| 61 | 0 | 0 | 0 | 0 | 0 | 0 | 0 | 0 |
| 62 | 0 | 0 | 0 | x | 0 | 0 | x | 0 |
| 63 | 0 | 0 | 0 | 0 | 0 | 0 | 0 | 0 |
| 64 | 0 | x | 0 | 0 | 0 | 0 | 0 | 0 |
| 65 | 0 | 0 | 0 | 0 | 0 | 0 | 0 | 0 |
| 66 | 0 | 0 | 0 | 0 | 0 | 0 | 0 | 0 |

1. **Overview of all feedback loops**


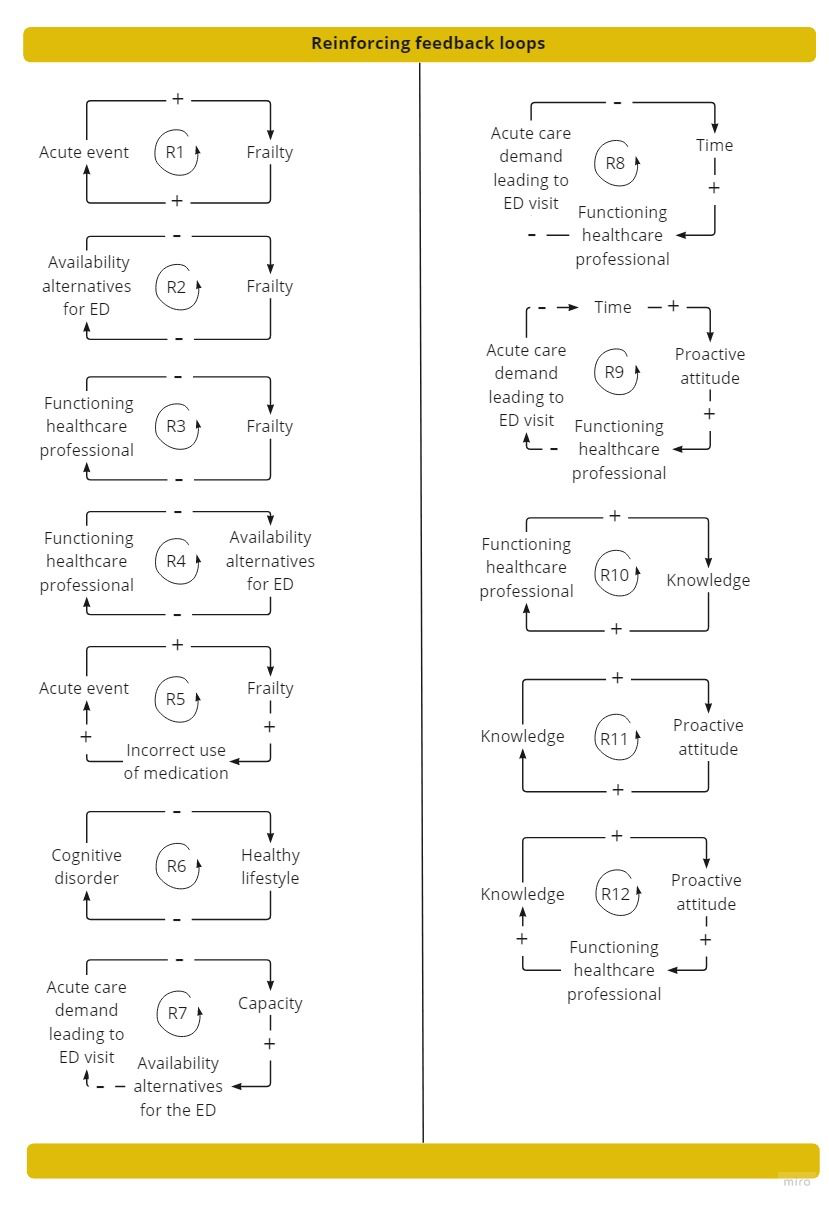


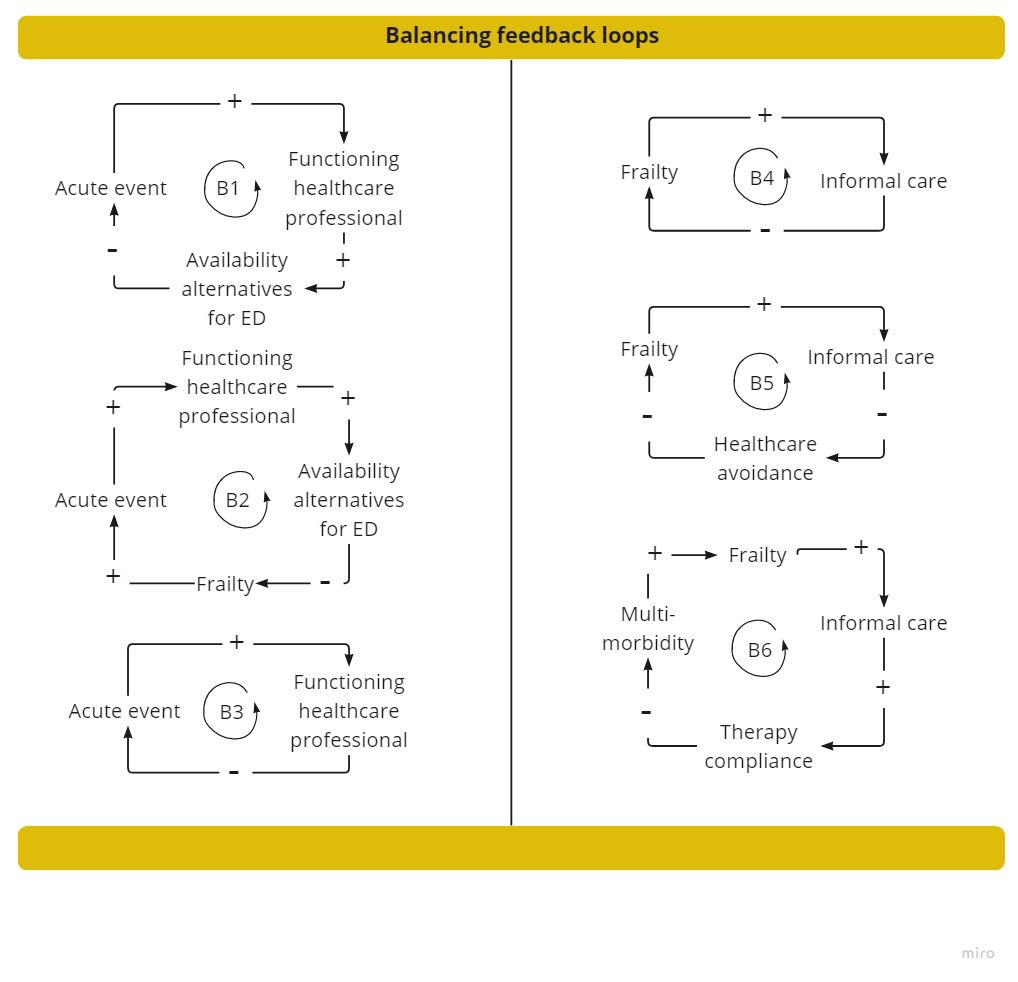


References

1. Samaras N, Chevalley T, Samaras D *et al.* Older patients in the emergency department: a review. *Ann Emerg Med* 2010;**56**:261–9.

2. Aminzadeh F, Dalziel WB. Older adults in the emergency department: a systematic review of patterns of use, adverse outcomes, and effectiveness of interventions. *Ann Emerg Med* 2002;**39**:238–47.

3. Schrijver E, Toppinga Q, de Vries O *et al.* An observational cohort study on geriatric patient profile in an emergency department in the Netherlands. *Netherlands journal of medicine* 2013;**71**:324–30.

4. Hohl CM, Dankoff J, Colacone A *et al.* Polypharmacy, adverse drug-related events, and potential adverse drug interactions in elderly patients presenting to an emergency department. *Ann Emerg Med* 2001;**38**:666–71.

5. Rutschmann O, Chevalley T, Zumwald C *et al.* Pitfalls in the emergency department triage of frail elderly patients without specific complaints. *Swiss Med Wkly* 2005.

6. O’Cathain A, Knowles E, Turner J *et al.* Explaining variation in emergency admissions: a mixed-methods study of emergency and urgent care systems. *Health Services and Delivery Research* 2014;**2**:1–126.

7. Halm EA, Lee C, Chassin MR. Is volume related to outcome in health care? A systematic review and methodologic critique of the literature. *Ann Intern Med* 2002;**137**:511–20.

8. Gandjour A, Lauterbach KW. The Practice-Makes-Perfect Hypothesis in the Context of Other Production Concepts in Health Care. *https://doi.org/101177/106286060301800407* 2003;**18**:171–5.

9. Luft HS, Hunt SS, Maerki SC *et al.* The volume-outcome relationship: practice-makes-perfect or selective-referral patterns? *Health Serv Res* 1987;**22**:157.

10. Lutz BJ, Hall AG, Vanhille SB *et al.* A Framework Illustrating Care-Seeking Among Older Adults in a Hospital Emergency Department. *Gerontologist* 2018;**58**:942–52.

11. Driesen BEJM, Merten H, Wagner C *et al.* Unplanned return presentations of older patients to the emergency department: A root cause analysis. *BMC Geriatr* 2020;**20**, DOI: 10.1186/s12877-020-01770-x.

12. Bennett HQ, Norton S, Bunn F *et al.* The impact of dementia on service use by individuals with a comorbid health condition: A comparison of two cross-sectional analyses conducted approximately 10 years apart. *BMC Med* 2018;**16**, DOI: 10.1186/s12916-018-1105-8.

13. Etters L, Goodall D, Harrison BE. Caregiver burden among dementia patient caregivers: A review of the literature. *J Am Acad Nurse Pract* 2008;**20**:423–8.

14. van den Broek S, Heiwegen N, Verhofstad M *et al.* Preventable emergency admissions of older adults: An observational mixed-method study of rates, associative factors and underlying causes in two Dutch hospitals. *BMJ Open* 2020;**10**, DOI: 10.1136/bmjopen-2020-040431.

15. Newcom Research & Consultancy. Werkdruk huisarts bedreigt kwaliteit zorg. (Dutch) https://www.newcom.nl/2018-werkdruk-huisarts/ (last accessed 1 June 2022). 2018.

16. Verhaegh M, Snijders F, Janssen L *et al.* Perspectives on the preventability of emergency department visits by older patients. *Netherlands Journal of Medicine* 2019;**77**:330–7.

17. Kolk D, Kruiswijk AF, MacNeil-Vroomen JL *et al.* Older patients’ perspectives on factors contributing to frequent visits to the emergency department: a qualitative interview study. *BMC Public Health* 2021;**21**, DOI: 10.1186/s12889-021-11755-z.

18. Sir Ö, Hesselink G, Schoon Y *et al.* Dutch emergency physicians insufficiently educated in geriatric emergency medicine: results of a nationwide survey. *Age Ageing* 2021;**50**:1997–2003.

19. Balakrishnan MP, Herndon JB, Zhang J *et al.* The Association of Health Literacy with Preventable ED Visits: A Cross-Sectional Study. *Acad Emerg Med* 2017;**24**:1042.

20. Greene JC, Haun JN, French DD *et al.* Reduced Hospitalizations, Emergency Room Visits, and Costs Associated with a Web-Based Health Literacy, Aligned-Incentive Intervention: Mixed Methods Study. *J Med Internet Res 2019;21(10):e14772 https://www.jmir.org/2019/10/e14772* 2019;**21**:e14772.

21. Ionescu-Ittu R, McCusker J, Ciampi A *et al.* Continuity of primary care and emergency department utilization among elderly people. *CMAJ : Canadian Medical Association journal* 2007;**177**:1362–8.

22. de Gelder J, Lucke JA, de Groot B *et al.* Predictors and Outcomes of Revisits in Older Adults Discharged from the Emergency Department. *J Am Geriatr Soc* 2018;**66**:735–41.

23. Dufour I, Chiu Y, Courteau J *et al.* Frequent emergency department use by older adults with ambulatory care sensitive conditions: A population-based cohort study. *Geriatr Gerontol Int* 2020;**20**, DOI: 10.1111/GGI.13875.

24. Chamberlain AM, Rutten LJF, Jacobson DJ *et al.* Multimorbidity, functional limitations, and outcomes: Interactions in a population-based cohort of older adults. *J Comorb* 2019;**9**, DOI: 10.1177/2235042X19873486.

25. Rostamzadeh A, Stapels J, Genske A *et al.* Health Literacy in Individuals at Risk for Alzheimer’s Dementia: A Systematic Review. *J Prev Alzheimers Dis* 2020;**7**:47–55.

26. Droogsma E, Van Asselt DZB, Scholzel-Dorenbos CJM *et al.* Nutritional status of community-dwelling elderly with newly diagnosed Alzheimer’s disease: prevalence of malnutrition and the relation of various factors to nutritional status. *J Nutr Health Aging* 2013;**17**:606–10.

27. Arlt S, Lindner R, Rösler A *et al.* Adherence to medication in patients with dementia: predictors and strategies for improvement. *Drugs Aging* 2008;**25**:1033–47.

28. Dufour I, Chouinard MC, Dubuc N *et al.* Factors associated with frequent use of emergency-department services in a geriatric population: A systematic review. *BMC Geriatr* 2019;**19**, DOI: 10.1186/s12877-019-1197-9.

29. Valtorta NK, Moore DC, Barron L *et al.* Older Adults’ Social Relationships and Health Care Utilization: A Systematic Review. *Am J Public Health* 2018;**108**:e1–10.

30. Patiëntenfederatie. Zorg dat medische gegevens altijd beschikbaar zijn. (dutch) https://www.patientenfederatie.nl/actueel/nieuws/zorg-dat-medische-gegevens-altijd-beschikbaar-zijn (Last accessed 1 june 2022). 2021.

31. Lie D, Carter-Pokras O, Braun B *et al.* What Do Health Literacy and Cultural Competence Have in Common? Calling for a Collaborative Pedagogy. *J Health Commun* 2012;**17**:13.

32. Degan TJ, Kelly PJ, Robinson LD *et al.* Health literacy in people living with mental illness: A latent profile analysis. *Psychiatry Res* 2019;**280**, DOI: 10.1016/J.PSYCHRES.2019.112499.

33. Dhana K, Evans DA, Rajan KB *et al.* Healthy lifestyle and the risk of Alzheimer dementia: Findings from 2 longitudinal studies. *Neurology* 2020;**95**:e374.

34. García-Pérez LE, Álvarez M, Dilla T *et al.* Adherence to Therapies in Patients with Type 2 Diabetes. *Diabetes Therapy* 2013;**4**:175.

35. Unverzagt S, Meyer G, Mittmann S *et al.* Improving Treatment Adherence in Heart Failure: A Systematic Review and Meta-analysis of Pharmacological and Lifestyle Interventions. *Dtsch Arztebl Int* 2016;**113**:423.

36. Burns JR, Musa P. Structural Validation of Causal Loop Diagrams. *In: Atlanta SD Conference*. 2001, 1–13.
